# Frequency of Severity of Disability in Patients with Grade III Lumbar Neural Foraminal Stenosis on Magnetic Resonance Imaging

**DOI:** 10.7759/cureus.4386

**Published:** 2019-04-04

**Authors:** Qazi Saad B Khalid, Raza Sayani, Muhammad Zeeshan, Shahbaz Alam

**Affiliations:** 1 Radiology, Dallah Hospital, Dallah, SAU; 2 Radiology, Aga Khan University Hospital, Karachi, PAK; 3 Radiology, Royal Blackburn Hospital / East Lancashire Hospital Trust (ELHT), Manchester, GBR

**Keywords:** lumbar neural foraminal stenosis, degenerative lumbar spinal stenosis, oswestry disability index, back pain

## Abstract

Background

Low back pain is a common condition and carries substantial socioeconomic implications. Magnetic resonance imaging (MRI) is the imaging modality of choice with lumbar neural foraminal stenosis being one of the most common causes of lower back pain syndromes. Studies have shown a lack of correlation between patients’ severity of disability and radiologically determined nerve root constriction. Therefore, the goal of this study will be to determine the frequency of severity of disability in patients with severe (i.e., grade III) lumbar neural foraminal stenosis on MRI to ascertain the impact of MRI diagnosis on clinical outcomes.

Materials and methods

Two hundred fifty patients of either gender with a history of backache referred for MRI were included by purposive sampling. Of these 250 patients, 27 patients had grade II lumbar neural foraminal stenosis, and 21 had grade I neural foraminal stenosis on MRI and were excluded. Thirty-two patients had a spinal infection (e.g., tuberculosis), and 24 patients had a history of trauma. Further, 31 patients were having follow-up scans for previously diagnosed lumbar neural foraminal stenosis. Hence, after excluding these cases, 115 patients were enrolled in this cross-sectional study with grade III lumbar neural foraminal stenosis on MRI.

Results

The mean age was 51 years (range: 20 to 82 years). Most of the patients (55.6%) were older than 50 years. The most common site of grade III lumbar neural foraminal stenosis was L4-L5 (56.5%). According to the Oswestry disability index, 47 patients (40.9%) had a severe disability, 32 (27.8%) had a moderate disability, 16 (13.9%) were diabled, 14 (12.2%) had a mild disability, and six (5.2%) were bedridden.

Conclusions

While MRI is the imaging modality of choice in degenerative lumbar spinal stenosis, clinical disabilities can be more extensive than what radiological findings may indicate in approximately 40% of the cases. Therefore, lumbar spinal stenosis should be a neuro-radiological diagnosis, and surgical decisions should be based on clinical scenarios in addition to MRI findings.

## Introduction

Low back pain is highly prevalent and has substantial socioeconomic implications [1]. It is the second most common concern encountered by primary care physicians (after the common cold). Up to 80% of all individuals will experience low back pain at some point in their lives. Unfortunately, a specific diagnosis is not made in 80% of low back pain cases [2]. Previous studies have reported a lifetime prevalence of low back pain of 65% to 80% [3]. In a local study by Siddiqui et al., the most common cause of low back pain was nerve root compression due to neural foraminal stenosis with a reported prevalence of 73% [4]. The management of patients with low back pain is either surgical or conservative based on the presence and severity of nerve root compression [5].

Magnetic resonance imaging (MRI) of the lumbar spine is the modality of choice for diagnosing nerve root compression in patients with low back pain with a reported sensitivity and specificity of 80.65% and 100%, respectively [6]. However, the degree of neural foramen constriction necessary for the symptoms of lumbar spinal stenosis to appear has not been established. Furthermore, the frequency of clinical symptoms (i.e., the patient’s disability) and the degree of a radiologically verified constriction have also not been clearly defined [7].

Previous studies present conflicting data between anatomic abnormalities of the lumbar spine detected via MRI, clinical history, and patient outcomes. A high prevalence of spine abnormalities was detected via MRI even in asymptomatic patients [1]. These incidental findings might lead to additional testing and carry the potential for unnecessary interventions, increased cost of care, and possibly worse outcomes. One such study shows 15.8% of total patients showing mild disability, 20.6% moderate disability, 39.6% severe disability, 19.04% crippled, and 5% of patients bedridden [7]. Therefore, it is important to determine the frequency of severity of disability in patients with neural foraminal stenosis diagnosed via MRI in our clinical settings.

This study aims to determine the frequency of severity of patients’ disability as calculated by the Oswestry disability index (ODI) in patients with grade III lumbar neural foraminal stenosis diagnosed on MRI. The study will then ascertain the impact of MRI diagnosis on clinical outcomes compared with careful patient and treatment selection based on clinical scenarios, which may result in reduced diagnoses, unnecessary surgeries, and financial and psychological stress.

## Materials and methods

This descriptive, cross-sectional study with non-probability, purposive sampling was conducted in the department of radiology for six months. Patients of either gender who were older than 20 years with grade III lumbar neural foraminal stenosis on MRI were included. Patients with a previous history of lumbar spine surgery and those who were getting follow-up scans with already diagnosed nerve root compression were excluded.

Informed consent was obtained. All information was collected through a designed proforma which included the Oswestry disability index (ODI)--a questionnaire containing six statements (denoted levels 0 to 5) in each of the 10 sections related to impairments like pain and disabilities such as personal care, lifting, walking, sitting, standing, sleeping, sex life, social life, and traveling. In each section, the patient chose the statement that best described his or her status. ODI is a simple, condition-specific, and preferred multidimensional tool because patients can easily comprehend the form, it is a self-assessment, and it encompasses a wide domain of functions, pain, and limitations as health status [7].

Patients with a score of 0% to 20% disability were considered minimally disabled, meaning patients can cope with most living activities. For most minimally disabled patients, no treatment is indicated apart from advice on lifting, sitting, and exercise. A score of 21% to 40% meant patients were classified as moderately disabled, and the patient experiences more pain and difficulty with sitting, lifting, and standing. Travel and social life are more difficult, and personal care, sexual activity, and sleeping are not grossly affected. These patients can generally be managed by conservative means. A score of 41% to 60% meant patients were considered severely disabled. Severely disabled patients have increased pain intensity that impacts the routines of daily life. These patients require a detailed investigation with the possibility for surgical correction. A score of 61% to 80% meant the patient was disabled, and back pain impinged on all aspects of the patient’s life, requiring positive intervention. Finally, a score of 81% to 100% meant low back pain was so severe that the patient became bedridden.

Date of examination, gender and any previous history of trauma were documented by credentialed MRI technician at the time of examination. Strict exclusion criteria were maintained to control the confounding variables of preexisting trauma or infection.

A Siemens Magnetom 1.5-tesla (76×18 channels; slice thickness, 4.0 mm; gap, 0.4 mm) MRI (Siemens AG, Munich, Germany) was used. The MRI radiographic images were interpreted by one experienced radiologist with five years of clinical experience. The patient’s disability level was then calculated by calculating the total ODI score, dividing by 50 and multiplying by 100 to calculate the percentage. The frequencies of each level of disability in cases of grade III lumbar neural foraminal stenosis were then calculated accordingly. The statistical analysis was done using IBM SPSS Statistics for Windows, Version 19.0 (IBM Corp, Armonk, NY). The frequencies and percentages for categorical variables like age, gender, and severity of patients’ disability were calculated. Furthermore, mean and standard deviation for the continuous variables (e.g., age) were also calculated. Stratification was performed with regards to age, gender, and severity of disability to see the effect of the variable on outcomes.

## Results

**Figure 1:**
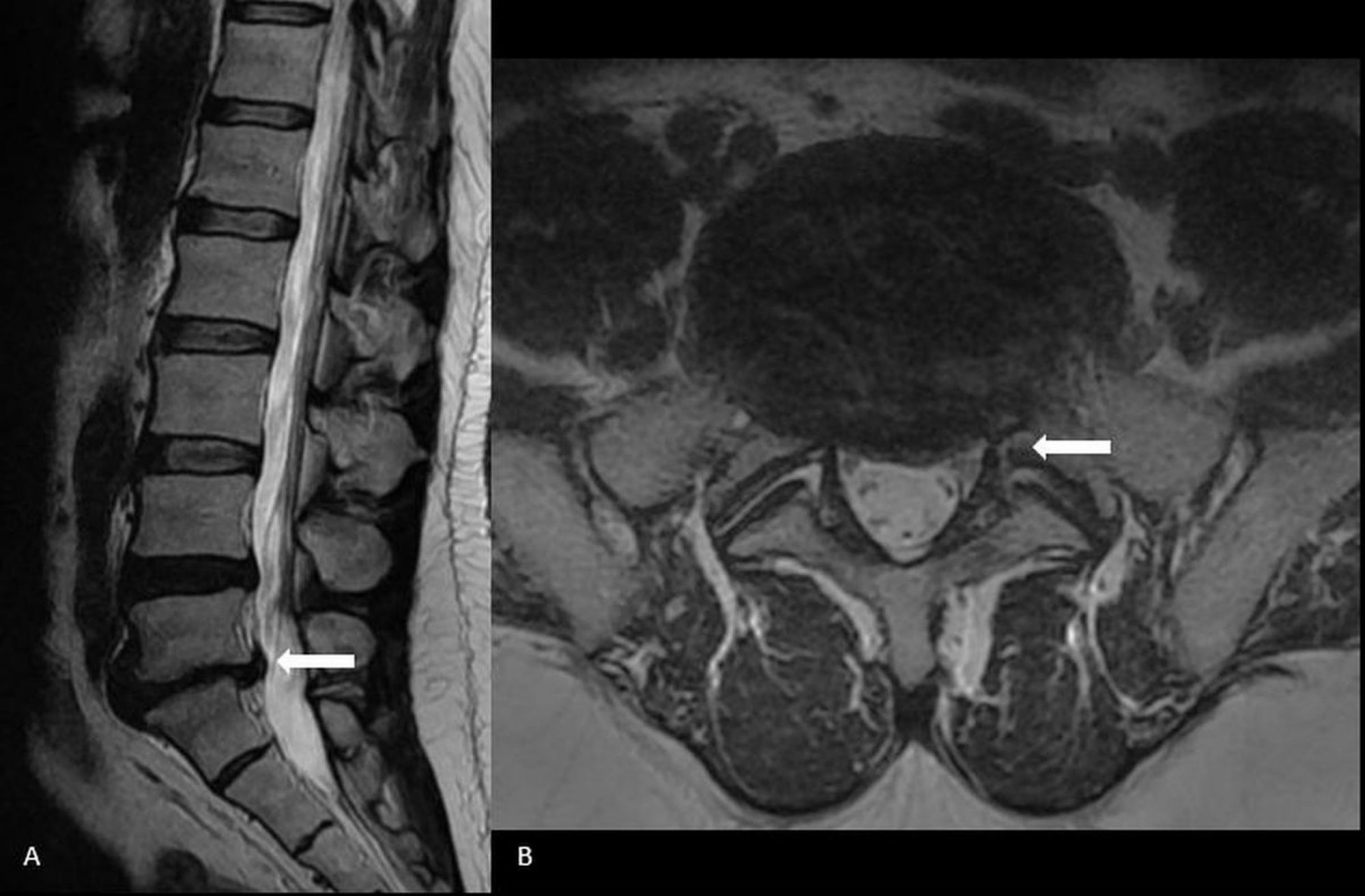
L5-S1 disc bulge with moderate thecal sac compression (arrow) on (A) Sagittal and (B) Axial plane.

A total of 250 patients who presented with backache and had MRI examination were included. Of these 250 patients, 27 patients had grade II lumbar neural foraminal stenosis, and 21 had grade I neural foraminal stenosis on MRI and were thus excluded. Thirty-two patients had a spinal infection (e.g., tuberculosis), and 24 patients had a history of trauma. Furthermore, 31 patients were having a follow-up scan for previously diagnosed lumbar neural foraminal stenosis. Hence, after excluding these cases, 115 patients with MRI-diagnosed grade III lumbar neural foraminal stenosis were included in the study (Figure 1).

There were 67 men (58.3%) and 48 (41.7%) women. The mean patient age was 51 (range, 20 to 82 years). Most patients (n=34; 29.6% were older than 60 years. Thirty patients (26.1%) were aged between 50 and 59 years, 31 (27%) were aged between 40 and 49 years, 11 (9.6%) patients were aged between 30 and 39 years, and nine (7.8%) were aged between 20 and 29 years.

Grade III lumbar neural foraminal stenosis was most common at the L4-L5 level (n=65; 56.5%), followed by the L5-S1 level (n=37;32.2%), the L3-L4 level (n=9; 7.8%), and the L2-L3 level (n=4; 3.5%).

Most cases of stenosis (n=45; 39.1%) involved the left side, 40 cases (34.8%) were bilateral, and 30 (26.1%) were on the right side. According to the ODI scores, 47 patients (40.9%) had a severe disability, 32 (27.8%) had moderate disability, 16 (13.9%) were disabled, 14 (12.2%) had a mild disability, and six (5.2%) were bedridden.

## Discussion

Low back pain is a common health condition affecting approximately 80% of people at some time in their lives. It is also the most prevalent cause of disability in people under age 45 and has significant socioeconomic implications. Unfortunately, a specific diagnosis cannot be made in approximately 80% of cases [8]. The most common cause for low back pain is nerve root compression due to neural foraminal stenosis [4].

Management options for low back pain may be mainly either surgical or conservative however long term outcome appear similar in both cases [9]. Surgery intends to decompress the area of narrowing determined on the basis of imaging so as to alleviate pressure on the nerves. This makes accurate analysis important for correct therapy selection [10].

MRI is the imaging modality of choice because of its excellent ability to provide great soft tissue delineation. However, the grade of compression on the thecal sac which is expected to be symptomatic for lumbar spinal stenosis is still not clear. Furthermore, the relationship between the clinical presentation and the imaging findings is still not established. Therefore, it is essential to see the frequency of severity of disability in patients with lumbar neural foraminal stenosis on MRI.

In our study, 69 of 115 patients (60%) had severe or more than severe disability scores as per the severity of foraminal stenosis seen on MRI. However, the remaining patient population (40%) showed only mild to moderate disability, far fewer than expected based on radiological assessments. Other studies found similar discrepancies between radiological appearance and clinical symptoms [7,11].

Preoperative evaluation was done by Sirvanci et al. in 63 patients with degenerative spinal stenosis [7]. No definite correlation was seen between radiological stenosis and ODI percentage. Eight patients with severe central stenosis and nine patients with moderate lateral stenosis on imaging demonstrated only minimal disability on ODI scores.

Amundsen et al. [11] evaluated 100 patients with backache radiologically and found different types of stenosis; however, no definite relationships could be identified. The imaging findings were much more than expected from the clinical presentation.

Hurri et al. [12] reported a significant correlation between stenosis and ODI percentage, but no such correlation could be recognized in other studies, including those by Jonsson et al. [13], Beattie et al. [14], and Lohman et al. [15]. Moreover, several studies including those by Boden et al. [16] and Szpalski et al. [17] have shown significant lumbar spinal abnormalities on MRI even in completely asymptomatic patients, further proving a lack of correlation between radiologically verified anatomical constriction and clinical picture.

Several theories may explain such discrepancies between the clinical symptoms and radiologically verified anatomical constrictions. One explanation is radiologist overcall; that is, radiologists label morphological changes which may be seen in normal healthy subjects as pathological. Another factor is the impact of positioning on anatomical constriction; extension and flexion movements can result in an increased or decreased cross-sectional diameter of the spinal canal [18]. As in most places, MRI is usually performed with the patient in the supine position; this can result in an underestimation of the spinal canal or foraminal stenosis on static imaging [19].

The most common intervertebral level involved in our patients was L4-L5, which aligned with the reports by other researchers [7, 20-21]. According to many studies the degenerative lumbar spinal stenosis becomes symptomatic usually in the fifth decade of life [22], which also aligns with our findings. We noted a continuous trend of increasing disability with respect to increasing age.

## Conclusions

Although MRI is the imaging modality of choice in cases of lumbar spinal stenosis because of its ability to provide excellent soft tissue resolution, no significant correlation could be demonstrated between imaging appearances and levels of severity of disability in patients with lumbar spinal stenosis in this study and previous studies. In our study, 40% of patients had only mild to moderate disability implying that their radiological changes were more extensive than expected from the clinical picture. Therefore, degenerative lumbar spinal stenosis is a clinico-radiological syndrome, and treatment decisions should incorporate radiological appearances in addition to the patient’s clinical picture before deciding between conservative or surgical management approaches.
